# New Oral Antitumor Drugs and Medication Safety in Uro-Oncology: Implications for Clinical Practice Based on a Subgroup Analysis of the AMBORA Trial

**DOI:** 10.3390/jcm11154558

**Published:** 2022-08-04

**Authors:** Katja Schlichtig, Lisa Cuba, Pauline Dürr, Laura Bellut, Norbert Meidenbauer, Frank Kunath, Peter J. Goebell, Andreas Mackensen, Frank Dörje, Martin F. Fromm, Bernd Wullich

**Affiliations:** 1 Institute of Experimental and Clinical Pharmacology and Toxicology, Friedrich-Alexander-Universität Erlangen-Nürnberg, 91054 Erlangen, Germany; 2 Comprehensive Cancer Center Erlangen-EMN, Erlangen University Hospital, 91054 Erlangen, Germany; 3 Pharmacy Department, Erlangen University Hospital, 91054 Erlangen, Germany; 4 Department of Urology and Pediatric Urology, Erlangen University Hospital, 91054 Erlangen, Germany; 5 Department of Internal Medicine 5, Hematology and Oncology, Erlangen University Hospital, 91054 Erlangen, Germany

**Keywords:** drug–related problems, medication errors, medication safety, prostate cancer, renal cell carcinoma

## Abstract

Oral antitumor therapeutics (OAT) bear a high risk for medication errors, e.g., due to drug–drug or drug–food interactions or incorrect drug intake. Advanced age, organ insufficiencies, and polymedication are putting uro-oncological patients at an even larger risk. This analysis sets out to (1) investigate the frequency and relevance of medication errors in patients with prostate cancer or renal cell carcinoma treated with OAT and (2) compile recommendations for clinical practice. This post-hoc subgroup analysis used data collected in the randomized AMBORA trial (2017–2020; DRKS00013271). Clinical pharmacologists/pharmacists conducted advanced medication reviews over 12 weeks after initiation of a new oral regimen and assessed the complete medication process for drug–related problems. Medication errors related to either the OAT, prescribed or prescription-free concomitant medication, or food were classified regarding cause and severity. We identified 67 medication errors in 38 patients within the complete medication within 12 weeks. Thereof, 55% were detected at therapy initiation, 27% were caused by the patients, and 25% were drug–drug or drug–food interactions. Problem-prone issues are summarized in a ‘medication safety table’ to provide recommendations for clinical practice in uro-oncology. Tailored strategies including intensified care by clinical pharmacologists/pharmacists should be implemented in clinical practice to improve medication safety.

## 1. Introduction

The systemic therapy of malignant tumors is becoming increasingly complex [1]. Several new oral antitumor therapeutics (OAT) were approved within the last decades and gain importance [2]. Patients and treatment teams often prefer OAT due to the convenient way of independent drug intake [3]. Nonetheless, OAT is associated with distinct challenges for medication safety [2,4,5]. Therapeutic success can be endangered by, e.g., drug–drug or drug–food interactions, limited patient adherence, complex dosing regimens, and toxicity [2,6,7,8]. Especially, patients with prostate cancer (PC) or renal cell carcinoma (RCC) are a high-risk population for medication errors (defined as ‘any preventable event that may cause or lead to inappropriate medication use or patient harm’ [9]) due to advanced age, organ insufficiencies, and polymedication [10].

To date, several pivotal clinical studies have shown significant improvements in progression-free or overall survival due to evolved treatment strategies for urological tumors (monotherapy or combinations, e.g., cabozantinib and nivolumab) [11]. Starting with the approval of the antihormonal agent abiraterone for metastatic PC, the tyrosine kinase inhibitors sunitinib and sorafenib have contributed to new treatment options for patients with metastatic RCC. Some of these new options can only be considered for individual patients after molecular analysis (e.g., olaparib in the presence of BRCA mutation). There are five new OAT approved for PC and eight for RCC (in Germany as of April 2022).

Data on the appropriate use of OAT in daily standard care within this high-risk population are rare. An analysis in a genitourinary oncology practice identified medication-related problems (e.g., side effects, drug–drug interactions, duplications) in 25% of 315 assessments performed by pharmacists in 20 patients [12].

An intensified clinical pharmacological/pharmaceutical care program added and integrated into the standard of care practice can considerably optimize the use of a broad range of approved new OAT as we could show in the recently published randomized AMBORA trial [13]. The systematic data collection and analysis using standard operating procedures within the AMBORA trial guaranteed almost complete demographic and medication data of excellent quality (e.g., advanced medication reviews, predefined time points over 12 weeks, consideration of the complete medication, see [14]). The trial results evidenced that medication errors, side effects, and patient-reported outcomes were significantly improved by an intensified clinical pharmacological/pharmaceutical care program compared to the usual standard of care. Importantly, the clinically highly relevant combined endpoint of severe side effects, treatment discontinuation, unscheduled hospital admissions, and death was reduced by 52% due to the intervention [13].

As detailed knowledge on, e.g., the causes and severity of medication errors in patients with PC or RCC treated with new OAT is still lacking, we now set out to (1) identify and describe especially problem-prone aspects for medication safety and (2) give tailored practical recommendations for healthcare professionals in uro-oncology.

## 2. Materials and Methods

### 2.1. Patients and Design

This analysis is based on the dataset of our previously published randomized, controlled, multicenter AMBORA trial [13,14] (Medication Safety with Oral Antitumor Therapy) and targets the subgroup of patients with PC or RCC in more detail. In brief, the AMBORA trial investigated the impact of an intensified clinical pharmacological/pharmaceutical care program on medication safety in 202 patients treated with new OAT independent of tumor entities. The term ‘new OAT’ was defined as drugs, which were approved after the first tyrosine kinase inhibitor imatinib in Germany since February 2001. Written informed consent was given by the patients prior to enrollment. Approval by the Ethics Committee of the Friedrich-Alexander-Universität Erlangen-Nürnberg was obtained and registration in the German Clinical Trials Register (DRKS00013271) was conducted. The study was carried out in Germany at 11 independent outpatient clinics and oncological practices (including one outpatient clinic for uro-oncology) associated with the CCC Erlangen-EMN.

Following a 1:1 randomization, patients were allocated between November 2017 and January 2020 to a control group (routine clinical care) or an intervention group (intensified clinical pharmacological/pharmaceutical care program). Over a follow-up period of 12 weeks per patient, clinical pharmacologists/pharmacists were involved in additional care sessions in the intervention group at predefined time points (at the initiation of the new oral regimen, week 0), and within the follow-up period (week 1, 4, and 12) and performed, e.g., patient counseling and medication management. For the present post-hoc subgroup analysis, patients with PC or RCC were retrieved from the dataset as published in [13] and the data were pooled from the control and the intervention group.

### 2.2. Data Collection

As previously described [13,14], the patient characteristics and the patients’ complete medications were inquired through structured patient interviews at the time point of initiation of the new oral regimen (baseline, week 0) and regularly updated within the follow-up counseling sessions after 4 and 12 weeks. Missing or unclear data was supplemented, e.g., by the patients’ electronic health records.

Besides demographic characteristics (e.g., age, gender), detailed clinical data (e.g., comorbidities, laboratory parameters) were collected. Patients were asked about habits [e.g., smoking, consumption of grapefruit (-products)] and the use of medication besides the OAT. The concomitant medication (prescribed drugs, Rx) and over-the-counter drugs (OTC, e.g., ibuprofen, herbal drugs, or dietary supplements) were explicitly inquired and taken into account by the clinical pharmacologist/pharmacist to ensure the best possible medication history (BPMH [15]). Topically administered drugs or specific drug intake routes (e.g., inhalatives, transdermal patches) were queried as well. Medication was classified according to the Anatomical Therapeutical Chemical (ATC) system code and the respective dosages and schedules were determined. For further analysis, the complete medication was divided into the subgroups ‘oral antitumor therapy’ or ‘co-medication’ as well as ‘prescribed’ or ‘over-the-counter’ drugs.

### 2.3. Medication Review

Clinical pharmacologists/pharmacists checked the complete medication process from prescribing to drug intake to detect medication errors [13]. They specifically checked whether the drugs were correctly and regularly handled by the patients. Advanced medication reviews [following the definition according to the Pharmaceutical Care Network Europe (PCNE [16])] when besides the patient interview, clinical data, and medication history is available as information were performed at each follow-up appointment. The medication history was screened using different tools and checklists [e.g., START/STOPP criteria [17], medication appropriateness index (MAI [18]), Beers Criteria [19]], and original research papers to identify (potential) medication errors. For each patient, the individual clinical situation was considered to rate whether the medication was appropriate.

At least two evidence-based drug interaction tools were utilized (Lexicomp^®^ Drug Interactions, Hudson, OH, USA [20] and Stockley’s Interactions Checker, London, UK [21] primarily for drug–drug interactions, the database of the Memorial Sloan Kettering Cancer Center, New York, NY, USA [22] on top for drug–herb or drug–food interactions). Following thorough medication analyses, every single medication error was judged and double-checked for clinical relevance by a second expert as previously described [13,14].

### 2.4. Typology of Medication Errors

The medication errors were categorized by the Pharmaceutical Care Network Europe (PCNE) classification tool (Version V8.02 [23]) that assigns, e.g., one problem and the underlying cause for each error. Medication errors were defined according to the National Coordinating Council for Medication Error Reporting and Prevention (NCC-MERP) as ‘any preventable event that may cause or lead to inappropriate medication use or patient harm’ [9]. According to the corresponding NCC-MERP algorithm [9], medication errors were allocated to categories for patient harm ranging from B (= did not reach the patient) to I (= patient death). For drug–drug or drug–food interactions, severity was documented according to Lexicomp^®^ [20]. Medication errors within the patients’ complete medication were further divided as to whether the OAT was involved, or the error occurred within the concomitant medication.

### 2.5. Medication Safety Table

In order to give practical recommendations regarding key medication safety issues with new oral uro-oncological regimens for healthcare professionals, a ‘medication-safety-table’ was created. The table specifically comprises new OAT currently approved for PC or RCC in Germany (as of April 2022). We took account of (1) the clinical experience from medication errors in patients with PC or RCC within the AMBORA trial (present analysis and [13,14]) and (2) key medication safety issues described in the German Summaries of Product Characteristics, databases (e.g., UpToDate^®^, Waltham, MA, USA [24]; CredibleMeds^®^, Tucson, AZ, USA [25]), as well as pertinent guidelines.

### 2.6. Statistical Analysis

For demographic and clinical patient characteristics, the medication history, and the typology of medication errors, descriptive statistics were applied and analyzed with Microsoft Excel^®^, Redmond, WA, USA. Frequency distributions were assessed by applying Pearson’s χ^2^-test and Fisher’s Exact test. Pairwise group differences were analyzed using unpaired *t*-tests or nonparametric Mann–Whitney U tests, and multigroup differences using the Jonckheere-Terpstra test with IBM SPSS Statistics 26.0, Ehningen, Germany (*p* values below 0.05 were considered statistically significant).

## 3. Results

### 3.1. Patients

The characteristics of the complete AMBORA population regarding demographics and medication use were previously published [13,14]. Of the 202 patients included in the AMBORA trial, 38 patients with PC or RCC were identified for this subgroup analysis. Detailed characteristics are presented in Table 1. Twenty (53%) and eighteen (47%) patients received OAT for PC and RCC, respectively. The patients were on average 70 years old and took a median number of 10 drugs at baseline (range: 2–21). Over-the-counter (OTC) drugs were regularly used by 34% (13/38) of the patients. The most frequently prescribed OAT were abiraterone (34%, 13/38) and cabozantinib (26%, 10/38). Patient baseline characteristics stratified for PC and RCC are shown in supplementary Table S1. A flow chart providing information on the allocation of patients with PC or RCC within the AMBORA trial including the numbers of medication errors stratified for control and intervention group is attached in supplementary Figure S1.

### 3.2. Medication Errors

#### 3.2.1. Numbers and Involved Medicines

**Figure 1:**
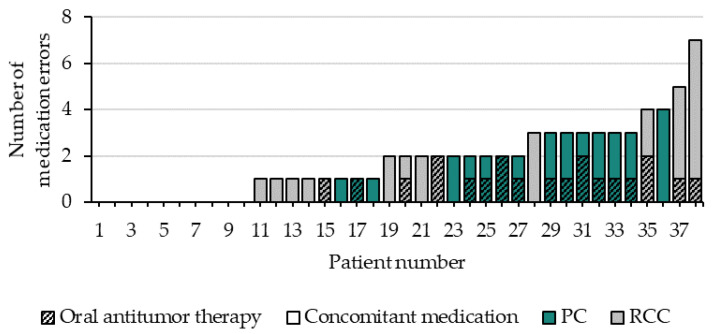
Number of medication errors in patients with PC or RCC treated with new oral antitumor drugs within the first 12 weeks of therapy stratified for the involved type of medication (oral antitumor therapy or concomitant medication) and the tumor entity. Abbreviations: PC = prostate cancer; RCC = renal cell carcinoma.

The data on all 335 medication errors detected within the entire AMBORA population were previously published [14]. The total number of 67 medication errors in the uro-oncological subgroup (1.8 medication errors per patient) within the first 12 weeks of treatment were detected in 74% (28/38) of the patients with PC or RCC (Figure 1). Forty-five percent of patients (17/38) had at least one medication error involving the OAT (Figure 1). The OAT was involved in 31% (21/67) of all errors. The average number of medication errors per patient was highest for olaparib (four errors in three patients). The number of medication errors per patient stratified for PC and RCC and group differences regarding demographic characteristics and the numbers of medication errors are presented in supplementary Table S2. No significant differences were found when comparing patients with PC and RCC, but the complete uro-oncological group (PC and RCC) was significantly older than the rest of the AMBORA population (*p* = 0.02). The most frequent therapeutic subgroups involved in all medication errors were analgesics, mineral supplements, antithrombotic agents, calcium channel blockers, lipid-modifying agents, and drugs for the treatment of bone diseases. The association of the number of all drugs at baseline with the number of medication errors related to the complete medication within the first 12 weeks of therapy is shown in supplementary Figure S2.

#### 3.2.2. Typology of Medication Errors

**Figure 2:**
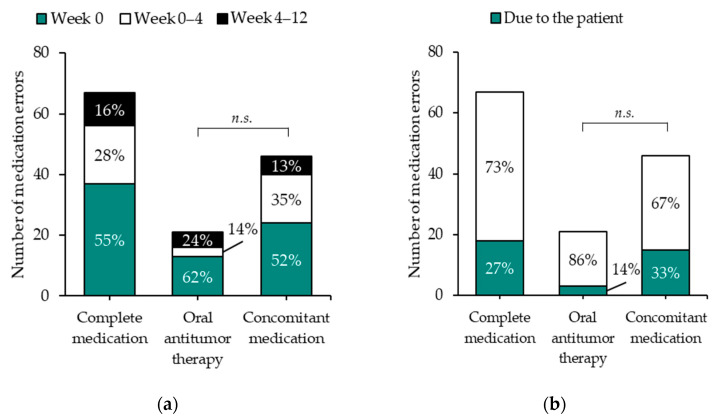

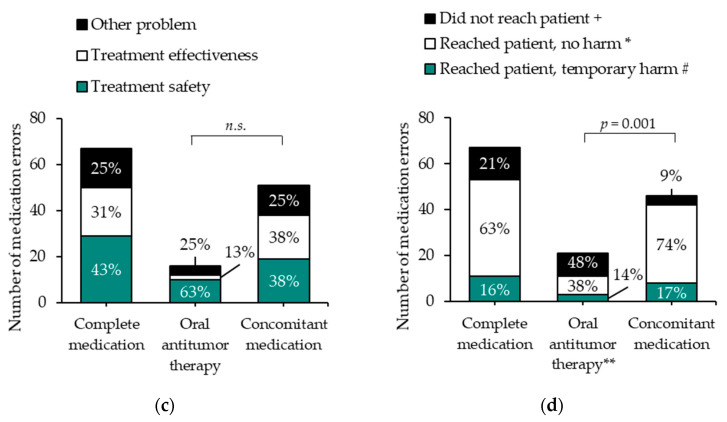
Number of medication errors in patients with PC or RCC treated with new oral antitumor drugs within the first 12 weeks of therapy stratified for (**a**) time point of occurrence; (**b**) patient as underlying cause, (**c**) type (anticipated consequence) of problem, and (**d**) harm categories according to the National Coordinating Council for Medication Error Reporting and Prevention (NCC-MERP index, [9]). Frequency distributions were analyzed using Pearson’s χ^2^-test and Fisher’s Exact test (*n.s.* = not significant). + This contains NCC-MERP category ‘B’. * This contains NCC-MERP categories ‘C’ and ‘D’. # This contains NCC-MERP categories ‘E’ to ‘I’. ** One patient suffered from constipation and inguinal hernia because he drank only half a glass of water per day, worrying to dilute abiraterone and thereby decrease therapeutic efficacy [14]; in another patient, therapy with nitrendipine was insufficient after he received enzalutamide (strong inducer of CYP3A4) resulting in hypertension; in the third case, the international normalized ratio (INR) of a patient under treatment with phenprocoumon decreased over time after therapy initiation of enzalutamide (strong inducer of CYP2C9) resulting in insufficient anticoagulation (INR 1.46 after 3 months).

Most medication errors (55%; 37/67) were detected immediately after the initiation of the OAT (week 0), 28% (19/67) within weeks 0 to 4, and 16% (11/67) within weeks 4 to 12 (Figure 2a). Twenty-seven percent of the medication errors (18/67) were caused by the patients (Figure 2b). The leading problem of all medication errors was ‘treatment safety’ (43%; 29/67) followed by ‘treatment effectiveness’ (31%; 21/67, Figure 2c). Treatment safety means a possible increased risk for toxicity (e.g., error may result in elevated plasma concentrations); treatment effectiveness means a possible reduced effect of the pharmacotherapy (e.g., error may result in decreased plasma concentrations). Most errors (63%; 42/67) reached the patient but did not cause harm (Figure 2d). Of the eleven errors that resulted in temporary harm, eight errors occurred within the co-medication and the OAT were involved in three errors (Figure 2d). The overall distribution of harm categories was significantly different when comparing medication errors related to the OAT with those that occurred within the concomitant medication (*p* = 0.001). In detail, 48% (10/21) of the medication errors involving the OAT did not reach the patients, whereas 9% (4/46) of the medication errors within the concomitant medication were detected before reaching the patients (Figure 2d).

Medication errors corresponding to the phase of the medication process are illustrated in Table 2, stratified for the causes according to the PCNE classification [23]. All errors involving the OAT stratified for each investigated drug are presented in supplementary Table S3. The highest prevalence of medication errors was found at the stage of prescribing (66%; 44/67) followed by drug use (33%; 22/67). A detailed description of the number and typology of medication errors stratified for PC and RCC is provided in supplementary Figures S3 and S4.

#### 3.2.3. Drug–Drug and Drug–Food Interactions

**Figure 3:**
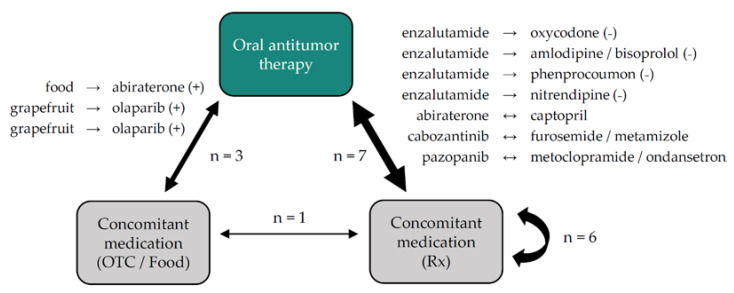
Number and types of drug–drug and drug–food interactions in patients with PC or RCC treated with new oral antitumor drugs within the first 12 weeks of therapy stratified for the involved type of medication. Arrows represent the ‘direction’ of the interactions as explained below: → = the ‘perpetrator’ drug on the left side of the arrow affects the ‘victim’ drug on the right side of the arrow leading to either: (+) = possible elevated toxicity of the ‘victim’ drug; (−) = possible reduced efficacy of the ‘victim’ drug; ↔ = mutual interactions requiring additional measures (e.g., blood pressure or ECG monitoring). Abbreviations: ECG = electrocardiogram; OTC = over-the-counter; Rx = prescribed drugs.

Within the complete medication, 17/67 (25%) drug–drug and drug–food interactions were detected, and the OAT was involved in 10/17 (59%). Within all pharmacokinetic drug–drug and drug–food interactions, the OAT was the ‘victim drug’ in three and the ‘perpetrator drug’ in four cases. A visual presentation of all pharmacokinetic and pharmacodynamic drug–drug and drug–food interactions with a focus on the OAT is given in Figure 3.

### 3.3. Medication Safety Table

**Figure 4:**
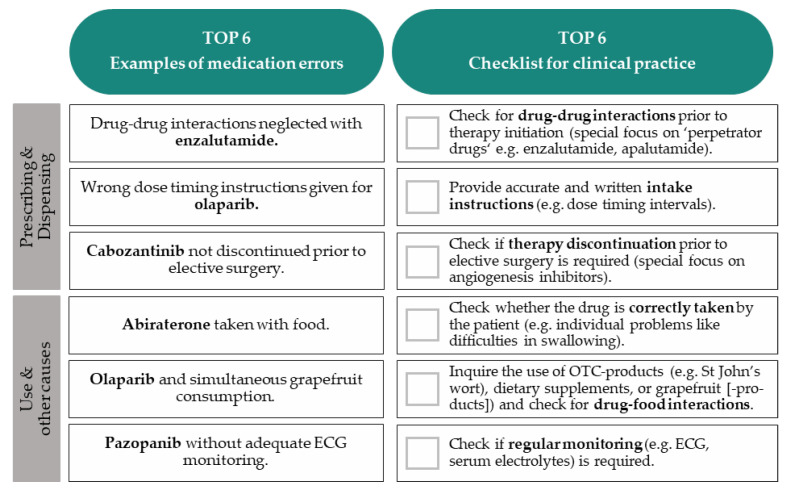
Top six medication errors in patients with PC or RCC treated with new oral antitumor drugs within the first 12 weeks of therapy and checklist for clinical practice. Abbreviations: ECG = electrocardiogram.

The developed supplementary medication safety table provides an overview including five new OAT for PC and eight for RCC. The chart specifies key medication safety issues, e.g., on (1) drug intake, (2) whether pharmacokinetics are affected by grapefruit (-products), St John’s wort, or gastric pH, (3) recommended dose adjustments in the case of organ insufficiency, and (4) monitoring (e.g., electrocardiograms). An excerpt with the top six medication errors involving the OAT and a resulting checklist for clinical practice is displayed in Figure 4.

## 4. Discussion

To the best of our knowledge, the present analysis is the first analysis of medication errors within 12 weeks after the initiation of a new OAT in patients with PC or RCC. In this subgroup analysis of the AMBORA trial, 74% of the patients with PC or RCC had at least one medication error within the complete medication, and 45% at least one involving the OAT. In our analysis, 55% of medication errors were detected at the initiation of the oral regimen, which turned out as being the most critical time point to detect medication errors [14]. An analysis of 249 patients treated with OAT for a broad spectrum of tumor entities in a Spanish tertiary hospital over a follow-up of six months reported a similar prevalence at baseline (68%) [26]. Furthermore, we detected a significantly higher number of medication errors involving the OAT before reaching the patients compared to errors related to the concomitant medication. Given the fact that patients were counseled right at initiation of the OAT, this finding underlines that systematic medication management can substantially help to prevent medication errors.

In line with Benoist et al., who reported a median number of eleven (range 1–26) co-medications in patients treated with enzalutamide [27], patients of the present subgroup analysis were treated with ten drugs (median, range 2–21). This number is higher compared to the entire AMBORA population [14] (median eight, range 1–67) and strengthens the assumption that patients with uro-oncological tumors represent a high-risk population due to polymedication. Thus, a detailed review of the complete medication is highly relevant as 69% of all errors occurred within the co-medication.

Several previous analyses on medication errors in uro-oncological patients investigated either single drugs, focused on drug–drug interactions only, or counted medication-related problems differently [7,12,27,28,29] and are therefore difficult to compare. In our analysis, drug–drug or drug–food interactions accounted only for 25% of all medication errors. Errors that cannot be detected by drug–drug interaction databases (e.g., drug handling, monitoring) should not be neglected. New OAT for PC or RCC are heterogeneous drugs with varying pharmacokinetic interaction profiles (supplementary medication safety table). Enzalutamide and apalutamide, both potent inducers of the CYP3A4 drug-metabolizing enzyme as well as other enzymes/transporters important for drug disposition [27,30], act as ‘perpetrator drugs’ and can lead to a loss of therapeutic effects of a variety of substances when administered simultaneously. Subsequently, enzalutamide, for example, has been reported to have a higher drug interaction potential (85% of patients [27]) compared to abiraterone (20–40% [31]), which is in line with our findings.

Inappropriate (co-)medication can not only cause side effects and thus affect quality of life, it can also diminish desired therapeutic outcome. A decreased overall survival by 4.6 months (8.0 vs. 12.6 months) was reported when pazopanib (pH-dependent solubility) was co-administered with proton-pump inhibitors compared to nonusers [32].

Furthermore, the absorption of several OAT is affected in a clinically relevant extent by concurrent food intake [33]. Thus, strict instructions regarding time intervals between drug and food intake need to be followed in five uro-oncological OAT. For example, abiraterone plasma concentrations are elevated up to 10-fold (AUC) depending on the fat content of the nutrition [34]. Detailed medication reviews including the use of OTC products (e.g., proton pump inhibitors, antacids) and patients’ food habits combined with appropriate counseling is crucial as 27% of all medication errors were caused by the patients themselves.

Sixteen percent of all errors resulted in temporary patient harm like adverse drug reactions. Whereas angiogenesis inhibitors are known to cause typical side effects such as bleeding complications, impaired wound healing, or elevated blood pressure [35], antiandrogen therapies may provoke a prolongation of the QT interval and increase the risk of Torsades de pointes arrhythmia [36]. Hence, interruption of treatment is recommended in case of surgical intervention under angiogenetic therapy [37] and regular electrocardiograms should be performed in most patients with androgen deprivation therapy to prevent patient harm (supplementary medication safety table).

The limitations of the present analysis are almost congruent with the previously published overall analysis of medication errors in the AMBORA trial [14]: Our data pooled both arms of the randomized, controlled trial [13]. Thus, the intervention in about 50% of the patients led to a partial prevention of medication errors over time. Overall, 35 and 32 medication errors were detected within this subgroup in the control and the intervention group, respectively. Furthermore, our analysis generates knowledge only about the first three months of treatment. Thus, no conclusions can be drawn regarding the effect of medication errors on hard clinical outcomes (e.g., the effectiveness of the OAT and/or disease progression). More research is required about medication errors in a larger patient cohort with a longer follow-up. Last, the overall number of patients was too small to generate further knowledge about risk-factors within this subgroup.

## 5. Conclusions

Medication errors throughout the medication process are frequent in patients with PC or RCC treated with new OAT. Independent drug intake, polymedication, and OAT with heterogeneous drug–drug and drug–food interaction profiles require specific measures that should be applied to these high-risk patients and the treatment teams to improve medication safety. Specific attention should be given to systematic checks for drug–drug or drug–food interactions (e.g., prescribed and prescription-free co-medication), accurate providing of instructions (e.g., intake with or without food), and appropriate monitoring (e.g., electrocardiograms).

In line with the findings of our previously published randomized AMBORA trial, this subgroup analysis indicates that the implementation of systematic medication management and patient counseling in routine clinical care performed by clinical pharmacologists/pharmacists can (1) prevent and resolve medication errors, (2) substantially improve medication safety, and (3) optimize outcomes in uro-oncological patients treated with new oral antitumor drugs.

## Figures and Tables

**Table 1:** Baseline clinical characteristics of the patients with PC or RCC.

Characteristic	No. (%) Total (N = 38)
**Age, years** (mean, range)	69.9 (47–85)
**Male sex**	36 (94.7)
**Female sex**	2 (5.3)
**ECOG status**	
0	10 (26.3)
1	19 (50.0)
>1	9 (23.7)
**Cancer type and oral anticancer therapy**	
**Prostate cancer**	20 (52.6)
Abiraterone	13 (34.2)
Enzalutamide	4 (10.5)
Olaparib	3 (7.9)
**Renal cell carcinoma**	18 (47.4)
Cabozantinib	10 (26.3)
Pazopanib	5 (13.2)
Sunitinib	2 (5.3)
Axitinib	1 (2.6)
**Anticancer regimen**	
Monotherapy * (oral)	37 (97.4)
Combination (oral + parenteral)	1 (2.6)
In-label	35 (92.1)
Off-label	3 (7.9)
**Number of all drugs**^#^ (median, range)	
Oral anticancer therapy	1 (1–1)
Concomitant medication	9 (1–20)
Complete medication	10 (2–21)
**Use of OTC drugs and habits**	
Yes	13 (34.2)
No	25 (65.8)
Consumption of grapefruit (-products)	6 (15.8)
**Comorbidities (Top 5)**	
Hypertension	28 (73.7)
Diabetes mellitus	10 (26.3)
Chronic renal failure	5 (13.2)
Dyslipidemia	5 (13.2)
Coronary heart disease	4 (10.5)
Glaucoma	4 (10.5)
Hypothyroidism	4 (10.5)
Atrial fibrillation	3 (7.9)

Patient characteristics of the complete AMBORA population were previously published [13,14]. * Thirteen patients received the combination of abiraterone (oral anticancer therapy) and prednisolone (co-medication). ^#^ Number of all drugs includes, e.g., oral, parenteral, topical, transdermal, inhalative, and OTC drugs. Abbreviations: ECOG = Eastern Cooperative Oncology Group; OTC = over-the-counter; PC = prostate cancer; RCC = renal cell carcinoma.

**Table 2:** Number of medication errors in patients with PC or RCC on new oral antitumor drugs classified by PCNE V8.02 [23].

Cause of Medication Errors			No. (%)		
			Complete Medication	Oral Antitumor Therapy	Co- Medication
**Prescribing**	Drug selection	Inappropriate drug according to guidelines/formulary	4 (6.0)	-	4 (6.0)
		Inappropriate drug (within guidelines, otherwise contraindicated)	2 (3.0)	-	2 (3.0)
		No indication for drug	2 (3.0)	-	2 (3.0)
		Inappropriate combination	11 (16.4)	7 (10.4)	4 (6.0)
		Inappropriate duplication	-	-	-
		No drug treatment in spite of existing indication	4 (6.0)	-	4 (6.0)
		Too many drugs prescribed for indication	-	-	-
	Drug form	Inappropriate drug form	4 (6.0)	1 (1.5)	3 (4.5)
	Dose selection	Drug dose too low	-	-	-
		Drug dose too high	3 (4.5)	-	3 (4.5)
		Dosage regimen not frequent enough	2 (3.0)	-	2 (3.0)
		Dosage regimen too frequent	4 (6.0)	1 (1.5)	3 (4.5)
		Dose timing instructions wrong, unclear or missing	7 (10.4)	4 (6.0) ^+^	3 (4.5)
	Treatment duration	Duration of treatment too short	-	-	-
		Duration of treatment too long	1 (1.5)	1 (1.5)	-
		**Total**	**44 (65.7)**	**14 (20.9)**	**30 (44.8)**
**Dispensing**	Dispensing	Prescribed drug not available	1 (1.5)	1 (1.5)	-
		Necessary information not provided	-	-	-
		Wrong drug, strength or dosage advised (OTC)	-	-	-
		Wrong drug or strength dispensed	-	-	-
		**Total**	**1 (1.5)**	**1 (1.5)**	**-**
**Use**	Drug use process	Not applicable in this trial ^a^	-	-	-
	Patient related	Patient uses/takes less drug than prescribed or does not take the drug at all	5 (7.5)	-	5 (7.5)
		Patient uses/takes more drug than prescribed	1 (1.5)	-	1 (1.5)
		Patient abuses drug	-	-	-
		Patient uses unnecessary drug	5 (7.5)	-	5 (7.5)
		Patient takes food that interacts ^b^	2 (3.0)	1 (1.5)	1 (1.5)
		Patient stores drug inappropriately	-	-	-
		Inappropriate timing or dosing intervals	3 (4.5)	-	3 (4.5)
		Patient uses the drug in a wrong way	2 (3.0)	2 (3.0) *	-
		Patient unable to use drug/form as directed	-	-	-
	Other	No or inappropriate outcome monitoring (incl. TDM)	1 (1.5)	1 (1.5)	-
		Other cause	3 (4.5)	2 (3.0) ^#^	1 (1.5)
		No obvious cause	-	-	-
		**Total**	**22 (32.8)**	**6 (9.0)**	**16 (23.9)**
**Total**			**67 (100)**	**21 (31.3)**	**46 (68.7)**

^a^ The cause domain ‘Drug use process’ was not applicable in this trial (patients getting the drug administered by a caregiver or healthcare professional were excluded from the AMBORA trial [13]. ^b^ This includes food, dietary supplements, and OTC drugs taken by the patient that interact with the medication. ^+^ e.g., olaparib thrice instead of twice daily. * e.g., abiraterone with apple sauce due to difficulties in swallowing. ^#^ e.g., cabazitaxel instead of cabozantinib documented in the medical record (sound-alike problem). Abbreviations: PCNE = Pharmaceutical Care Network Europe; TDM = therapeutic drug monitoring.

## Data Availability

Not applicable.

## Supplementary Materials

The following supporting information can be downloaded at: https://www.mdpi.com/article/10.3390/jcm11154558/s1, Figure S1: Flow chart of patient allocation of patients with PC or RCC treated with new oral antitumor drugs within the AMBORA trial [13] stratified for both tumor entities (green), intervention or control group, and the respective number of medication errors (grey); Figure S2: Association of the number of medication errors per patient related to the complete medication with the number of all drugs at baseline in patients with PC or RCC treated with new oral antitumor drugs within the first 12 weeks of therapy; Figure S3: Number of medication errors in patients with PC or RCC treated with new oral antitumor drugs within the first 12 weeks of therapy stratified for both tumor entities and (a) time point of occurrence, (b) patient as underlying cause; Figure S4: Number of medication errors in patients with PC or RCC treated with new oral antitumor drugs within the first 12 weeks of therapy stratified for both tumor entities and (a) type of problem, and (b) harm categories according to the National Coordinating Council for Medication Error Reporting and Prevention (NCC-MERP [9]); Supplementary medication safety table: Characteristics of new oral uro-oncological drugs; Table S1: Baseline clinical characteristics of the patients with PC or RCC stratified for both tumor entities; Table S2: Analysis of group differences regarding selected clinical characteristics and the number of medication errors per patient in patients treated with new oral antitumor drugs within the first 12 weeks of therapy stratified for the groups PC, RCC, and all other tumor entities of the AMBORA population; Table S3: Number of medication errors related to the oral antitumor therapy in patients with PC or RCC stratified for each oral antitumor drug and the respective cause according to PCNE V8.02 classification [23].

## References

[1] Zarrabi K., Paroya A., Wu S. (2019). Emerging therapeutic agents for genitourinary cancers. J. Hematol. Oncol..

[2] Schlichtig K., Dürr P., Dörje F., Fromm M.F. (2019). New oral anti-cancer drugs and medication safety. Dtsch. Arztebl. Int..

[3] Aisner J. (2007). Overview of the changing paradigm in cancer treatment: Oral chemotherapy. Am. J. Health-Syst. Pharm. AJHP Off. J. Am. Soc. Health-Syst. Pharm..

[4] Weingart S.N., Toro J., Spencer J., Duncombe D., Gross A., Bartel S., Miransky J., Partridge A., Shulman L.N., Connor M. (2010). Medication errors involving oral chemotherapy. Cancer.

[5] Zerillo J.A., Goldenberg B.A., Kotecha R.R., Tewari A.K., Jacobson J.O., Krzyzanowska M.K. (2018). Interventions to improve oral chemotherapy safety and quality: A systematic review. JAMA Oncol..

[6] Weingart S.N., Zhang L., Sweeney M., Hassett M. (2018). Chemotherapy medication errors. Lancet Oncol..

[7] van Leeuwen R.W.F., Brundel D.H.S., Neef C., van Gelder T., Mathijssen R.H.J., Burger D.M., Jansman F.G.A. (2013). Prevalence of potential drug-drug interactions in cancer patients treated with oral anticancer drugs. Br. J. Cancer.

[8] Greer J.A., Amoyal N., Nisotel L., Fishbein J.N., MacDonald J., Stagl J., Lennes I., Temel J.S., Safren S.A., Pirl W.F. (2016). A systematic review of adherence to oral antineoplastic therapies. Oncologist.

[9] Snyder R.A., Abarca J., Meza J.L., Rothschild J.M., Rizos A., Bates D.W. (2007). Reliability evaluation of the adapted national coordinating council medication error reporting and prevention (NCC MERP) index. Pharmacoepidemiol. Drug Saf..

[10] Spratt D.E., Shore N., Sartor O., Rathkopf D., Olivier K. (2021). Treating the patient and not just the cancer: Therapeutic burden in prostate cancer. Prostate Cancer Prostatic Dis..

[11] Choueiri T.K., Powles T., Burotto M., Escudier B., Bourlon M.T., Zurawski B., Oyervides Juárez V.M., Hsieh J.J., Basso U., Shah A.Y. (2021). Nivolumab plus cabozantinib versus sunitinib for advanced renal-cell carcinoma. N. Engl. J. Med..

[12] Holle L.M., Puri S., Clement J.M. (2016). Physician-pharmacist collaboration for oral chemotherapy monitoring: Insights from an academic genitourinary oncology practice. J. Oncol. Pharm. Pract. Off. Publ. Int. Soc. Oncol. Pharm. Pract..

[13] Dürr P., Schlichtig K., Kelz C., Deutsch B., Maas R., Eckart M.J., Wilke J., Wagner H., Wolff K., Preuß C. (2021). The randomized AMBORA trial: Impact of pharmacological/pharmaceutical care on medication safety and patient-reported outcomes during treatment with new oral anticancer agents. J. Clin. Oncol. Off. J. Am. Soc. Clin. Oncol..

[14] Schlichtig K., Dürr P., Dörje F., Fromm M.F. (2021). Medication errors during treatment with new oral anticancer agents: Consequences for clinical practice based on the AMBORA study. Clin. Pharmacol Ther..

[15] World Health Organisation: The High5s Project—Standard Operating Protocol for Medication Reconciliation Version 3 2014.

[16] Pharmaceutical Care Network Europe Foundation: PCNE Statement on Medication Review, V3 2013. https://www.pcne.org/upload/files/150_20160504_PCNE_MedRevtypes.pdf.

[17] O’Mahony D., O’Sullivan D., Byrne S., O’Connor M.N., Ryan C., Gallagher P. (2015). STOPP/START criteria for potentially inappropriate prescribing in older people: Version 2. Age Ageing.

[18] Hanlon J.T., Schmader K.E., Samsa G.P., Weinberger M., Uttech K.M., Lewis I.K., Cohen H.J., Feussner J.R. (1992). A method for assessing drug therapy appropriateness. J. Clin. Epidemiol..

[19] American Geriatrics Society (2019). American geriatrics society 2019 updated AGS Beers Criteria^®^ for potentially inappropriate medication use in older adults. J. Am. Geriatr. Soc..

[20] Lexicomp® Drug Interactions. https://online.lexi.com.

[21] Preston CL: Stockley’s Interactions Checker. London: Pharmaceutical Press. http://www.medicinescomplete.com/.

[22] Memorial Sloan Kettering Cancer Center: Search about Herbs. https://www.mskcc.org/cancer-care/diagnosis-treatment/symptom-management/integrative-medicine/herbs/search.

[23] Pharmaceutical Care Network Europe Foundation Classification for Drug Related Problems, V8.02 2003–2017. https://www.pcne.org/upload/files/230_PCNE_classification_V8-02.pdf.

[24] UpToDate®: Drug Informations. https://www.uptodate.com/login.

[25] CredibleMeds® QT Drugs Lists. https://www.crediblemeds.org.

[26] Ribed A., Romero-Jimenez R.M., Escudero-Vilaplana V., Iglesias-Peinado I., Herranz-Alonso A., Codina C., Sanjurjo-Saez M. (2016). Pharmaceutical care program for onco-hematologic outpatients: Safety, efficiency and patient satisfaction. Int. J. Clin. Pharm..

[27] Benoist G.E., van Oort I.M., Smeenk S., Javad A., Somford D.M., Burger D.M., Mehra N., van Erp N.P. (2018). Drug-drug interaction potential in men treated with enzalutamide: Mind the gap. Br. J. Clin. Pharmacol..

[28] Jamani R., Lee E.K., Berry S.R., Saluja R., DeAngelis C., Giotis A., Emmenegger U. (2016). High prevalence of potential drug-drug interactions in patients with castration-resistant prostate cancer treated with abiraterone acetate. Eur. J. Clin. Pharmacol..

[29] Kruse V., Somers A., Van Bortel L., De Both A., Van Belle S., Rottey S. (2014). Sunitinib for metastatic renal cell cancer patients: Observational study highlighting the risk of important drug-drug interactions. J. Clin. Pharm. Ther..

[30] Duran I., Carles J., Bulat I., Hellemans P., Mitselos A., Ward P., Jiao J., Armas D., Chien C. (2020). Pharmacokinetic drug-drug interaction of apalutamide, part 1: Clinical studies in healthy men and patients with castration-resistant prostate cancer. Clin. Pharm..

[31] Bonnet C., Boudou-Rouquette P., Azoulay-Rutman E., Huillard O., Golmard J.L., Carton E., Noé G., Vidal M., Orvoen G., Chah Wakilian A. (2017). Potential drug-drug interactions with abiraterone in metastatic castration-resistant prostate cancer patients: A prevalence study in France. Cancer Chemother Pharm..

[32] Mir O., Touati N., Lia M., Litière S., Le Cesne A., Sleijfer S., Blay J.Y., Leahy M., Young R., Mathijssen R.H.J. (2019). Impact of concomitant administration of gastric acid-suppressive agents and pazopanib on outcomes in soft-tissue sarcoma patients treated within the EORTC 62043/62072 trials. Clin. Cancer Res..

[33] Deng J., Brar S.S., Lesko L.J. (2018). To take or not to take with meals? Unraveling issues related to food effects labeling for oral antineoplastic drugs. Clin. Pharmacol. Drug Dev..

[34] Chi K.N., Spratlin J., Kollmannsberger C., North S., Pankras C., Gonzalez M., Bernard A., Stieltjes H., Peng L., Jiao J. (2015). Food effects on abiraterone pharmacokinetics in healthy subjects and patients with metastatic castration-resistant prostate cancer. J. Clin. Pharmacol..

[35] Fogli S., Porta C., Del Re M., Crucitta S., Gianfilippo G., Danesi R., Rini B.I., Schmidinger M. (2020). Optimizing treatment of renal cell carcinoma with VEGFR-TKIs: A comparison of clinical pharmacology and drug-drug interactions of anti-angiogenic drugs. Cancer Treat. Rev..

[36] Gagliano-Jucá T., Travison T.G., Kantoff P.W., Nguyen P.L., Taplin M.E., Kibel A.S., Huang G., Bearup R., Schram H., Manley R. (2018). Androgen deprivation therapy is associated with prolongation of QTc interval in men with prostate cancer. J. Endocr. Soc..

[37] Villanueva-Bueno C., Escudero-Vilaplana V., Collado-Borrell R., Giménez-Manzorro Á., Ribed A., Marzal-Alfaro B., Revuelta-Herrero J.L., Gonzalez-Haba E., Herranz A., Sanjurjo M. (2022). Medication guide for the perioperative management of oral antineoplastic agents in cancer patients. Expert Opin. Drug Saf..

