# Supplementary material for: New Oral Antitumor Drugs and Medication Safety in Uro-Oncology: Implications for Clinical Practice Based on a Subgroup Analysis of the AMBORA Trial
Source: J Clin Med. 2022 Aug 4;11(15):4558. doi: 10.3390/jcm11154558 (PMC9369799; doi:10.3390/jcm11154558)
Supplement: Supplementary file 1 [file jcm-11-04558-s001.zip › Figure_S1.pdf]

Figure S1. Flow chart of patient allocation of patients with PC or RCC treated with new oral antitumor drugs within the AMBORA trial [13] stratified for both tumor entities (green), intervention or control group, and the respective number of medication errors (grey).

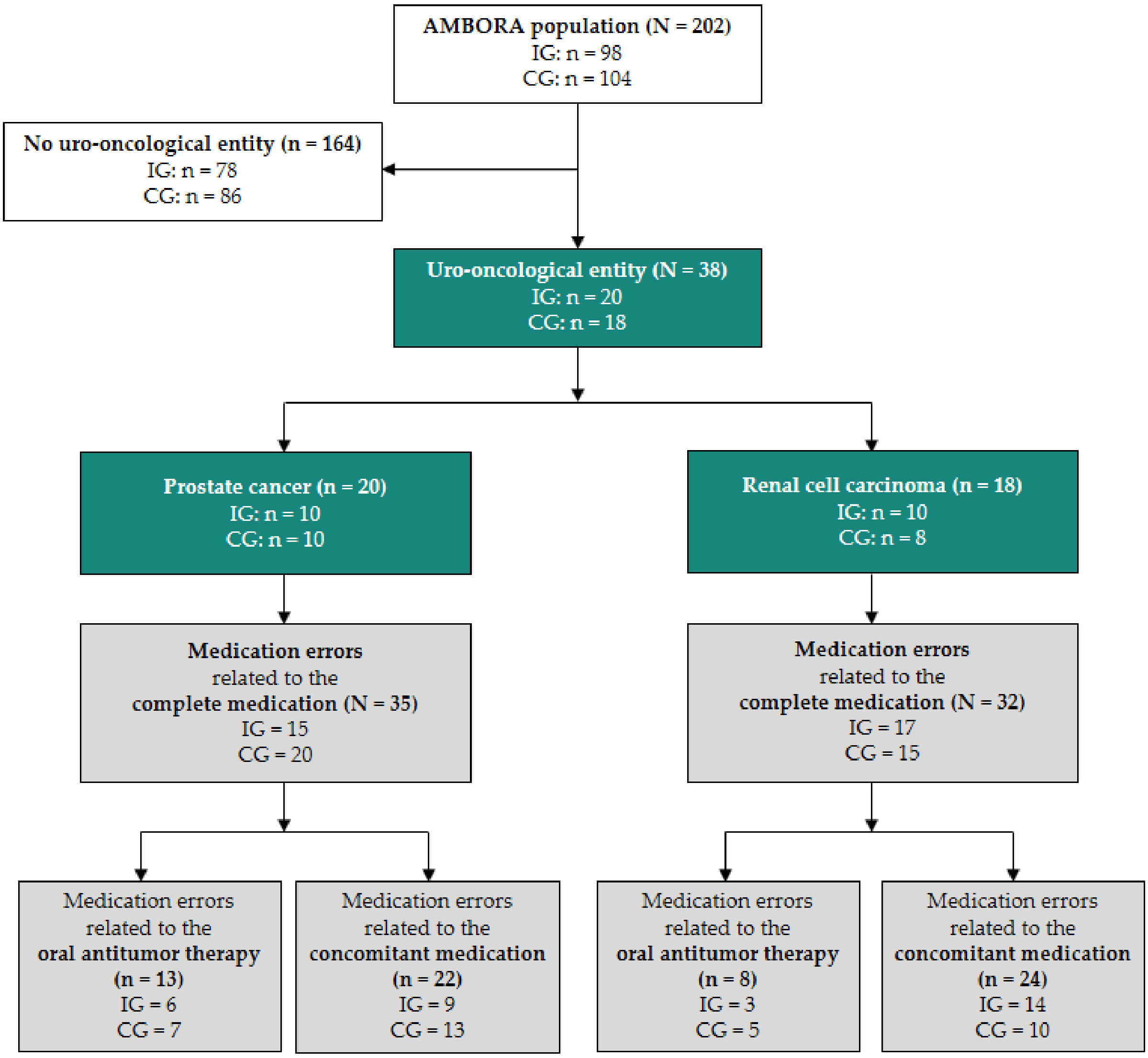

Abbreviations: CG = control group; IC = intervention group; PC = prostate cancer; RCC = renal cell carcinoma.
