# Supplementary material for: New Oral Antitumor Drugs and Medication Safety in Uro-Oncology: Implications for Clinical Practice Based on a Subgroup Analysis of the AMBORA Trial
Source: J Clin Med. 2022 Aug 4;11(15):4558. doi: 10.3390/jcm11154558 (PMC9369799; doi:10.3390/jcm11154558)
Supplement: Supplementary file 1 [file jcm-11-04558-s001.zip › Figure_S2.pdf]

Figure S2. Association of the number of medication errors per patient related to the complete medication with the number of all drugs at baseline in patients with PC or RCC treated with new oral antitumor drugs within the first 12 weeks of therapy.

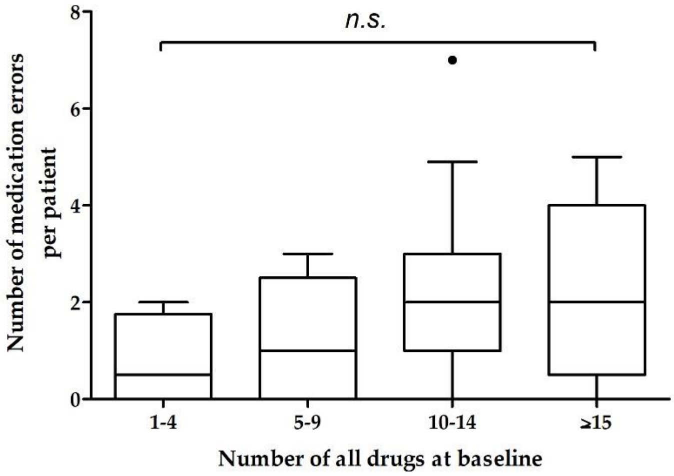

Box Plots with 10th and 90th percentiles. $p = 0.078$ (Jonckheere-Terpstra test).
Abbreviations: *n.s.* = not significant; PC = prostate cancer; RCC = renal cell carcinoma.
