# Supplementary material for: New Oral Antitumor Drugs and Medication Safety in Uro-Oncology: Implications for Clinical Practice Based on a Subgroup Analysis of the AMBORA Trial
Source: J Clin Med. 2022 Aug 4;11(15):4558. doi: 10.3390/jcm11154558 (PMC9369799; doi:10.3390/jcm11154558)
Supplement: Supplementary file 1 [file jcm-11-04558-s001.zip › Figure_S3.pdf]

Figure S3. Number of medication errors in patients with PC or RCC treated with new oral antitumor drugs within the first 12 weeks of therapy stratified for both tumor entities and (**a**) time point of occurrence, (**b**) patient as underlying cause.

(a)

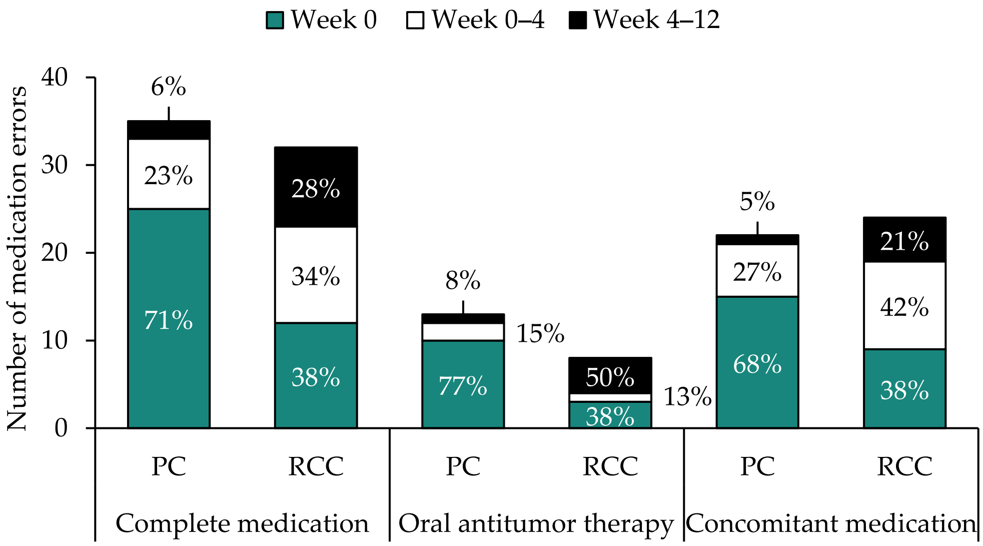

(b)

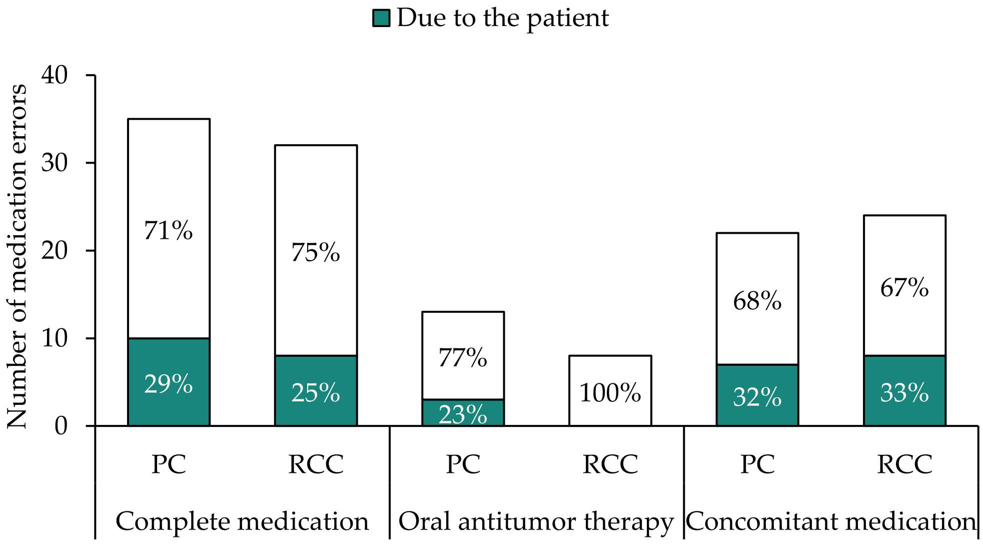

Abbreviations: PC = prostate cancer; RCC = renal cell carcinoma.
