# Supplementary material for: New Oral Antitumor Drugs and Medication Safety in Uro-Oncology: Implications for Clinical Practice Based on a Subgroup Analysis of the AMBORA Trial
Source: J Clin Med. 2022 Aug 4;11(15):4558. doi: 10.3390/jcm11154558 (PMC9369799; doi:10.3390/jcm11154558)
Supplement: Supplementary file 1 [file jcm-11-04558-s001.zip › Supplementary_medication_safety_table.pdf]

Supplementary medication safety table. Characteristics of new oral uro-oncological drugs.

		Dosing and Administration				Dose Adjustment		Surgery
	**Antitumor agent** Main target [1]	**Dosing** [1]	**Intake relative to food**[#] [1]	**Influence of food on AUC** [1]	**Dose missed (m) or vomited (v)** [1], [2]	**Renal impairment** [1], [3]	**Hepatic impairment**** [1], [3]	**Perioperative discontinuation***[#] [3]
**Renal Cell Carcinoma**	**Axitinib** VEGFR	5 mg BID	with / without	↓↑ (- 10 % with low-fat meal + 19 % with high-fat meal)	m, v: skip dose, next dose at regular time	< 15 ml/min: caution	moderate: 2 mg BID [1]; reduce by ~ 50 % [3] severe: avoid	2 days before / 2 weeks after
	**Cabozantinib** VEGFR	monotherapy: 60 mg OD combination with nivolumab: 40 mg OD	1 h before / 2 h after	↑ (+ 57 % with high-fat meal)	m: skip dose if next dose is due in less than 12 h v: n.a.	caution < 30 ml/min: avoid	moderate: monotherapy 40 mg OD, combination 20 mg OD [3] severe: avoid [1]	3 weeks before / 2 weeks after
	**Everolimus** mTOR	monotherapy: 10 mg OD combination with lenvatinib: 5 mg OD	with / without	↓ (- 32 % with low-fat meal, - 22 % with high-fat meal)	m: skip dose, next dose at regular time v: n.a.	not necessary	mild: 7.5 mg OD moderate: 5 mg OD severe: 2.5 mg OD (benefit / risk)	1 weeks before / 2 weeks after
	**Lenvatinib** VEGFR	Combination with everolimus: 18 mg OD; pembrolizumab: 20 mg OD	with / without	↔	m: skip dose if next dose is due in less than 12 h v: n.a.	< 30 ml/min: 10 mg OD	severe: 10 mg OD	1 weeks before / 2 weeks after
	**Pazopanib** VEGFR	800 mg OD	1 h before / 2 h after	↑ (+ 2-fold)	m: skip dose, next dose at regular time v: n.a.	< 30 ml/min: caution	moderate: 200 mg OD severe: avoid	1 week before / 2 weeks after
	**Sorafenib** VEGFR	400 mg BID	with / without high-fat meal: 1 h before / 2 h after	↓ (- 30 % with high-fat meal)	m: take it as soon as possible unless next dose is imminent v: n.a.	not necessary [1] < 40 ml/min: 200 mg BID [3]	moderate: 200 mg BID [3] severe: n.a.	10 days before / 2 weeks after
	**Sunitinib** VEGFR	50 mg OD for 4 weeks of a 6-week cycle	with / without	↔	m: skip dose, next dose at regular time v: n.a.	not necessary	severe: avoid	3 weeks before / 2 weeks after
	**Tivozanib** VEGFR	1340 µg OD on day 1 to 21 of a 28-day cycle	with / without	↔	m, v: skip dose, next dose at regular time	< 30 ml/min: caution [1] ≥ 15 ml/min: not necessary [3]	moderate: 1340 µg OD every second day [1]; 890 µg OD [3] severe: avoid [1]	24 days before / 2 weeks after
**Prostate Cancer**	**Abiraterone** CYP17	1000 mg OD (combination with prednisone/prednisolone)	1 h before / 2 h after	↑ (+ up to 10-fold)	m: skip dose, next dose at regular time v: n.a.	< 30 ml/min: caution [1] not necessary [3]	moderate: caution [1]; 250 mg OD [3] severe: contraindication [1]	n.a.
	**Apalutamide** androgen receptor	240 mg OD	with / without	↔	m: take missed dose on the same day v: n.a.	< 30 ml/min: caution	severe: avoid	n.a.
	**Darolutamide** androgen receptor	600 mg BID	with	↑ (+ 2- to 2,5-fold)	m: take dose as soon as noticed, but do not administer double doses v: n.a.	< 30 ml/min: 300 mg BID	moderate: 300 mg BID severe: 300 mg BID [1]; n.a. [3]	n.a.
	**Enzalutamide** androgen receptor	160 mg OD	with / without	↔	m: take missed dose on the same day v: n.a.	< 30 ml/min: caution	not necessary	n.a.
	**Olaparib (as tablet)** PARP	300 mg BID	with / without	↑ (+ 8 %)	m: skip dose, next dose at regular time v: n.a.	≤ 50 ml/min: 200 mg BID ≤ 30 ml/min: avoid	severe: avoid	n.a.

Note: Special recommendations are highlighted in the respective color. In case of discrepancies, both sources are shown in the panels. **Disclaimer**: Correctness, actuality and completeness of data can not be guaranteed despite diligent review. No liability is assumed for any errors or damages resulting from the information provided. **Abbreviations**: AUC = area under the curve; BID = twice daily; CYP = cytochrome P450; ECG = electrocardiogram; mTOR = mammalian target of rapamycin; n.a. = not available; OD = once daily; PARP = poly (ADP-ribose) polymerase; PPI = proton pump inhibitor; QTc = corrected QT time interval; TdP = torsade de pointes arrhythmia; VEGFR = vascular endothelial growth factor. **Legends:** ↓ = decreased; ↑ = increased; ↔ = not influenced; *reinitiation after clinical assessment of adequate wound healing. ** mild, moderate, or severe hepatic impairment is equivalent to Child-Pugh A, B, and C, respectively; [#]time is to be seen as the minimum time interval (at least X hours before / at least Y hours after). **References** (as of April 2022): [1] German Summary of Product Characteristics; [2] German Patient Information Leaflet; [3] UpToDate® Drug Information; [4] CredibleMeds® QT Drug List; [5] *National Comprehensive Cancer Network* (NCCN) Guidelines® Antiemesis Version 1.2022.

Supplementary medication safety table. Characteristics of new oral uro-oncological drugs.

		Drug-drug / drug-food interactions				Others		
	**Antitumor agent** Main target [1]	**Grapefruit (-products)** [1], [3]	**St John's wort** [1], [3]	**Substrate of CYP** [1]	**Gastric-pH-elevating drugs**** (PPI/H_2-antagonists/ antacids) [1], [3]	**Risk of TdP** [4]	**Prolongation of QTc interval** (recommendations e.g. regarding monitoring of serum electrolytes or ECG) [1], [3]	**Specific recommendations** (selection, e.g. antiemetic prophylaxis) [1], [3], [5]
**Renal Cell Carcinoma**	**Axitinib** VEGFR	avoid	avoid	3A4 (1A2) (2C19)	n.a.	n.a.	n.a.	n.a.
	**Cabozantinib** VEGFR	caution [1] avoid [3]	avoid	3A4 (2C9)	no influence	possible	electrolytes regularly [3] consider ECG monitoring [1]	moderate to high emetic risk: consider antiemetic regimen [3],[5]
	**Everolimus** mTOR	avoid	avoid	3A4	n.a.	n.a.	n.a.	consider Pneumocystis pneumonia prophylaxis [1] prophylaxis for mucositis [1]
	**Lenvatinib** VEGFR	n.a.	n.a.	(3A4)	n.a.	possible	calcium at least monthly, other electrolytes at baseline and regularly; ECG in risk patients	moderate to high emetic risk (≥ 12 mg OD): consider antiemetic regimen [3], [5]
	**Pazopanib** VEGFR	avoid	n.a. [1] monitor therapy [3]	3A4 (1A2) (2C8)	**PPI:** avoid **H_2-antagonists:** 10 h before / 2 h after pazopanib **antacids:** 2 h before / 1 h after pazopanib	possible	electrolytes and ECG in risk patients at baseline and regularly	tumor lysis syndrome: use with caution in risk patients [1,3]
	**Sorafenib** VEGFR	clinical relevance unlikely [1] n.a. [3]	decreased sorafenib plasma levels possible [1] monitor therapy [3]	(3A4)	n.a. [1] **PPI:** monitor therapy [3]	possible	electrolytes and ECG in risk patients at baseline and regularly	n.a.
	**Sunitinib** VEGFR	avoid [1] consider therapy modification [3]	avoid	3A4	n.a.	possible	electrolytes and ECG in risk patients (or when administered with strong CYP3A4 inhibitors) at baseline and regularly	tumor lysis syndrome: use with caution in risk patients [1,3]
	**Tivozanib** VEGFR	n.a.	contraindication [1] monitor therapy [3]	3A4	n.a.	n.a.	electrolytes and ECG in risk patients at baseline and regularly	n.a.
**Prostate Cancer**	**Abiraterone** CYP17	n.a.	avoid [1] monitor therapy [3]	3A4	n.a.	conditional	caution in patients with hypokalemia [1] monitor at least monthly for hypokalemia [3]	CYP2C8, CYP2D6 inhibitor [1]
	**Apalutamide** androgen receptor	n.a.	n.a.	2C8 (3A4)	no influence	possible	clinical risk assessment [1] no recommendations for monitoring	increased risk for falls and fractures: consider bone-modifying agents [1], [3] CYP3A4 inducer, CYP2C8 inhibitor [1]
	**Darolutamide** androgen receptor	n.a.	avoid	3A4	n.a.	possible	clinical risk assessment no recommendations for monitoring [1] n.a. [3]	n.a.
	**Enzalutamide** androgen receptor	n.a.	n.a. [1] avoid [3]	2C8 (3A4)	n.a.	possible	clinical risk assessment no recommendations for monitoring [1] n.a. [3]	CYP3A4, CYP2C9, CYP2C19 inducer [1]
	**Olaparib (as tablet)** PARP	avoid	avoid	3A4	n.a.	n.a.	n.a.	antiemetic prophylaxis not necessary [1] moderate to high emetic risk: consider antiemetic regimen [5]

Note: Special recommendations are highlighted in the respective color. In case of discrepancies, both sources are shown in the panels. **Disclaimer**: Correctness, actuality and completeness of data can not be guaranteed despite diligent review. No liability is assumed for any errors or damages resulting from the information provided. **Abbreviations**: AUC = area under the curve; BID = twice daily; CYP = cytochrome P450; ECG = electrocardiogram; mTOR = mammalian target of rapamycin; n.a. = not available; OD = once daily; PARP = poly (ADP-ribose) polymerase; PPI = proton pump inhibitor; QTc = corrected QT time interval; TdP = torsade de pointes arrhythmia; VEGFR = vascular endothelial growth factor. **Legends:** ↓ = decreased; ↑ = increased; ↔ = not influenced; *reinitiation after clinical assessment of adequate wound healing. ** mild, moderate, or severe hepatic impairment is equivalent to Child-Pugh A, B, and C, respectively; ‡time is to be seen as the minimum time interval (at least X hours before / at least Y hours after). **References** (as of April 2022): [1] German Summary of Product Characteristics; [2] German Patient Information Leaflet; [3] UpToDate® Drug Information; [4] CredibleMeds® QT Drug List; [5] *National Comprehensive Cancer Network* (NCCN) Guidelines® Antiemesis Version 1.2022.
