# Supplementary material for: New Oral Antitumor Drugs and Medication Safety in Uro-Oncology: Implications for Clinical Practice Based on a Subgroup Analysis of the AMBORA Trial
Source: J Clin Med. 2022 Aug 4;11(15):4558. doi: 10.3390/jcm11154558 (PMC9369799; doi:10.3390/jcm11154558)
Supplement: Supplementary file 1 [file jcm-11-04558-s001.zip › Table_S1.pdf]

Table S1. Baseline clinical characteristics of the patients with PC or RCC stratified for both tumor entities.

Characteristic	No. (%) PC (n = 20)	No. (%) RCC (n = 18)	No. (%) Total (N = 38)
**Age, years** (mean, range)	69.5 (49 - 85)	70.3 (47 - 81)	69.9 (47 - 85)
**Male sex**	20 (100.0)	16 (88.9)	36 (94.7)
**Female sex**	-	2 (11.1)	2 (5.3)
**ECOG status**			
0	6 (30.0)	4 (22.2)	10 (26.3)
1	10 (50.0)	9 (50.0)	19 (50.0)
> 1	4 (20.0)	5 (27.8)	9 (23.7)
**Cancer type and oral anticancer therapy**			
**Prostate cancer**	20 (100.0)	-	20 (52.6)
Abiraterone	13 (65.0)	-	13 (34.2)
Enzalutamide	4 (20.0)	-	4 (10.5)
Olaparib	3 (15.0)	-	3 (7.9)
**Renal cell carcinoma**	-	18 (100.0)	18 (47.4)
Cabozantinib	-	10 (55.6)	10 (26.3)
Pazopanib	-	5 (27.8)	5 (13.2)
Sunitinib	-	2 (11.1)	2 (5.3)
Axitinib	-	1 (5.6)	1 (2.6)
**Anticancer regimen**			
Monotherapy (oral)	7 (35.0)	17 (94.4)	24 (63.2)
Combination* (oral + oral)	13 (65.0)	-	13 (34.2)
Combination (oral + parenteral)	-	1 (5.6)	1 (2.6)
In-label	17 (85.0)	18 (100.0)	35 (92.1)
Off-label	3 (15.0)	-	3 (7.9)
**Number of all drugs**[#] (median, range)			
Oral anticancer therapy	1 (1 - 1)	1 (1 - 1)	1 (1 - 1)
Concomitant medication	9 (3 - 16)	9 (1 - 20)	9 (1 - 20)
Complete medication	10 (4 - 17)	10 (2 - 21)	10 (2 - 21)
**Use of OTC drugs and habits**			
Yes	8 (40.0)	5 (27.8)	13 (34.2)
No	12 (60.0)	13 (72.2)	25 (65.8)
Consumption of grapefruit (-products)	4 (20.0)	2 (11.1)	6 (15.8)
**Comorbidities (Top 5)**			
Hypertension	16 (80.0)	12 (66.7)	28 (73.7)
Diabetes mellitus	4 (20.0)	6 (33.3)	10 (26.3)
Chronic renal failure	-	5 (27.8)	5 (13.2)
Dyslipidemia	2 (10.0)	3 (16.7)	5 (13.2)
Coronary heart disease	2 (10.0)	2 (11.1)	4 (10.5)
Glaucoma	3 (15.0)	1 (5.6)	4 (10.5)
Hypothyroidism	1 (5.0)	3 (16.7)	4 (10.5)
Atrial fibrillation	2 (10.0)	1 (5.6)	3 (7.9)

Patient characteristics of the complete AMBORA population were previously published [13,14].
* All 13 patients received the combination of abiraterone (oral anticancer therapy) and prednisolone (co-medication)
[#] Number of all drugs includes e.g., oral, parenteral, topical, transdermal, inhalative, and OTC drugs
Abbreviations: ECOG = Eastern Cooperative Oncology Group; OTC = over-the-counter; PC = prostate cancer; RCC = renal cell carcinoma.
