# Supplementary material for: New Oral Antitumor Drugs and Medication Safety in Uro-Oncology: Implications for Clinical Practice Based on a Subgroup Analysis of the AMBORA Trial
Source: J Clin Med. 2022 Aug 4;11(15):4558. doi: 10.3390/jcm11154558 (PMC9369799; doi:10.3390/jcm11154558)
Supplement: Supplementary file 1 [file jcm-11-04558-s001.zip › Table_S2.pdf]

Table S2. Analysis of group differences regarding selected clinical characteristics and the number of medication errors per patient in patients treated with new oral antitumor drugs within the first 12 weeks of therapy stratified for the groups PC, RCC, and all other tumor entities of the AMBORA population.

	Mean (SD)			Mean (SD)		
**Characteristic**	**PC (n = 20)**	**RCC (n = 18)**	***p***	**Uro-oncological subgroup (N = 38)**	**AMBORA all other tumor entities (N = 164)**	***p***
**Age, years**	69.5 (9.7)	70.3 (9.2)	*n.s.**	69.9 (9.4)	65.1 (12.0)	*0.02**
**ECOG status**	1.0 (0.8)	1.1 (0.7)	*n.s.**	1.0 (0.8)	0.8 (0.7)	*n.s.**
**Number of all drugs**[#]						
Complete medication (median, range)	10 (4 - 17)	10 (2 - 21)	*n.s.***	10 (2 - 21)	8 (1 - 67)	*n.s.***
**Number of medication errors per patient**						
Involving the OAT	0.7 (0.7)	0.4 (0.7)	*n.s.**	0.6 (0.7)	0.6 (0.8)	*n.s.**
Within the concomitant medication	1.1 (1.1)	1.3 (1.7)	*n.s.**	1.2 (1.4)	1.0 (1.2)	*n.s.**
Within the complete medication	1.8 (1.3)	1.8 (1.9)	*n.s.**	1.8 (1.6)	1.6 (1.6)	*n.s.**
**Number of medication errors due to the patient**						
Within the complete medication	0.5 (0.9)	0.4 (0.7)	*n.s.**	0.5 (0.8)	0.5 (0.8)	*n.s.**

Patient characteristics of the complete AMBORA population were previously published [13,14].
[#] Number of all drugs includes e.g. oral, parenteral, topical, transdermal, inhalative, and OTC drugs.
* Unpaired t-test (two-tailed).
** Nonparametric Mann-Whitney-U-test.
Abbreviations: ECOG = Eastern Cooperative Oncology Group; *n.s.* = not significant; OTC = over-the-counter; OAT = oral antitumor therapy; PC = prostate cancer; RCC = renal cell carcinoma; SD = standard deviation.
