# Supplementary material for: New Oral Antitumor Drugs and Medication Safety in Uro-Oncology: Implications for Clinical Practice Based on a Subgroup Analysis of the AMBORA Trial
Source: J Clin Med. 2022 Aug 4;11(15):4558. doi: 10.3390/jcm11154558 (PMC9369799; doi:10.3390/jcm11154558)
Supplement: Supplementary file 1 [file jcm-11-04558-s001.zip › Table_S3.pdf]

Table S3. Number of medication errors related to the oral antitumor therapy in patients with PC or RCC stratified for each oral antitumor drug and the respective cause according to PCNE V8.02 classification [23].

			**Total No. (%)**	**PC No. (%)**			**RCC No. (%)**			
		**Causes of medication errors**	**Oral antitumor therapy**	**Abiraterone**	**Enzalutamide**	**Olaparib**	**Axitinib**	**Cabozantinib**	**Pazopanib**	**Sunitib**
**Prescribing**	Drug selection	Inappropriate combination	7 (33.3)	1 (4.8)	4 (19.0)	-	-	1 (4.8)	1 (4.8)	-
	Drug form	Inappropriate drug form	1 (4.8)	1 (4.8)	-	-	-	-	-	-
	Dose selection	Dosage regimen too frequent	1 (4.8)	-	-	-	-	-	1 (4.8)	-
		Dose timing instructions wrong, unclear or missing	4 (19.0)	1 (4.8)	-	3 (14.3)	-	-	-	-
	Treatment duration	Duration of treatment too long	1 (4.8)	-	-	-	-	1 (4.8)	-	-
**Dispensing**	Dispensing	Prescribed drug not available	1 (4.8)	-	-	-	1 (4.8)	-	-	-
**Use**	Patient related	Patient takes food that interacts [a]	1 (4.8)	-	-	1 (4.8)	-	-	-	
		Patient uses the drug in a wrong way	2 (9.5)	2 (9.5)	-	-	-	-	-	-
	Other	No or inappropriate outcome monitoring (incl. TDM)	1 (4.8)	-	-	-	-	-	1 (4.8)	-
		Other cause	2 (9.5)	-	-	-	-	2 (9.5)	-	-
		**Total number of medication errors**	**21 (100.0)**	**5 (23.8)**	**4 (19.0)**	**4 (19.0)**	**1 (4.8)**	**4 (19.0)**	**3 (14.3)**	**-**
		Total number of patients with ≥ 1 medication error	***17***	***5***	***3****	***3****	***1***	***3****	***2****	***-***
		Total number of patients with respective OAT	***38***	***13***	***4***	***3***	***1***	***10***	***5***	***2***

[a] This includes food, dietary supplements, and over-the-counter (OTC) drugs taken by the patient that interact with the medication.
* One patient with two medication errors.
For better visual clarity, only the respective PCNE causes of medication errors involving the oral antitumor drug are displayed in the table.
Abbreviations: OAT = oral antitumor therapy; PC = prostate cancer; PCNE = Pharmaceutical Care Network Europe; RCC = renal cell carcinoma; TDM = therapeutic drug monitoring.
